# Melanoma Risk in 12,205 Kidney Transplant Recipients Receiving Calcineurin Inhibitor-Based Immunosuppression: A Nationwide Analysis of Polish National Health Fund Data (2010–2022)

**DOI:** 10.3390/cancers18040642

**Published:** 2026-02-16

**Authors:** Aleksandra Kulbat, Wojciech M. Wysocki, Mateusz Kulbat, Marta Kołodziej-Rzepa, Tomasz Wojewoda

**Affiliations:** 1 Chair of Surgery, Faculty of Medicine and Health Sciences, Andrzej Frycz Modrzewski Krakow University, 30-705 Krakow, Poland; 2 Department of Oncological Surgery, 5th Military Clinical Hospital in Krakow, 30-901 Krakow, Poland; 3 Maria Sklodowska-Curie National Research Institute of Oncology, 00-001 Warsaw, Poland; 4 Department of Othopedy and Traumatology, 5th Military Clinical Hospital in Krakow, 30-901 Krakow, Poland

**Keywords:** calcineurin inhibitors, cyclosporine, tacrolimus, skin cancer, melanoma skin cancer, renal transplant recipients

## Abstract

**Background**: Numerous studies have demonstrated the impact of post-transplant immunosuppressive therapy on cancer development, particularly in the skin. Despite the growing number of observations, there is still a lack of comprehensive analyses assessing the influence of specific immunosuppressive regimens on the development of melanoma in kidney transplant recipients. The aim of this study was to describe the incidence and temporal patterns of cutaneous melanoma (CM) among kidney transplant recipients in Poland between 2010 and 2022, with particular focus on recipients treated with calcineurin inhibitor (CNI)-based immunosuppression, including cyclosporine (CsA) and tacrolimus (TAC). **Methods**: This nationwide, descriptive analysis was conducted using comprehensive national administrative data obtained from the Polish National Health Fund (PNHF) and the Polish National Cancer Registry (PNCR). The study assessed the incidence of cutaneous melanoma and temporal trends over the study period. **Results**: Melanoma skin cancer occurred in 27 cases observed in a population of 12,205 kidney transplant recipients over 13 years of follow-up, corresponding to a final cumulative incidence of 0.23% (95% CI: 0.15–0.34). Calcineurin inhibitor (CNI)-based regimens in kidney transplant recipients were associated with significantly lower risk profiles over 10 years, with only nine cases observed in a cohort of 7107 patients, culminating in a cumulative incidence of 0.13% (95% CI: 0.06–0.24). The stratified analysis of CM incidence under CNI-based immunosuppressive regimens by calcineurin inhibitor type revealed a modest cumulative risk in the TAC subgroup, reaching 0.14% (95% CI: 0.06–0.26) by year 10, with all nine observed cases occurring exclusively in this group, while the CsA subgroup reported zero events throughout the follow-up period. Risk differences progressively increased to 0.14% (95% CI: −0.20 to 0.26) by year 10, but they were not statistically significant in any year (*p* ≥ 0.230). **Conclusions:** Although all melanoma cases occurred in the TAC subgroup, the data do not allow us to conclude that TAC confers a higher risk than CsA. This lack of significance likely reflects both the rarity of events and the limited statistical power to detect small differences between TAC and CsA. These results highlight the need for careful dermatologic monitoring in all kidney transplant recipients, while the choice of calcineurin inhibitor should be individualized based on patient-specific factors.

## Simple Summary

Kidney transplant recipients have an increased risk of skin cancer due to long-term immunosuppressive therapy, but data on melanoma risk associated with specific immunosuppressive drugs remain limited. In this nationwide study, we analyzed the occurrence of cutaneous melanoma among kidney transplant recipients in Poland between 2010 and 2022 using national administrative databases. Melanoma was a rare event, affecting fewer than 1 in 400 transplant recipients over more than 10 years of follow-up. All observed melanoma cases occurred in patients treated with tacrolimus-based immunosuppression, while no cases were observed among patients receiving cyclosporine; however, the differences between treatment groups were not statistically significant due to the small number of events. These findings suggest that melanoma risk after kidney transplantation is low but underscore the importance of regular dermatologic surveillance in all transplant recipients, regardless of the specific calcineurin inhibitor used.

## 1. Introduction

Cutaneous melanoma (CM) is a relatively rare but aggressive form of skin malignancy with an increasing incidence worldwide. In the general population, melanoma incidence varies geographically and demographically, but rates have been rising over recent decades. Among solid organ transplant recipients, including kidney transplant recipients, melanoma risk is elevated relative to the general population, likely due to chronic immunosuppression. Analyses using large transplant registry data have demonstrated that the standardized incidence ratio (SIR) for invasive melanoma may be more than twofold higher in transplant recipients than in non-transplanted individuals (SIR = 2.20; 95% CI 2.01–2.39) [1]. Furthermore, recent meta-analytic evidence suggests that treatment with calcineurin inhibitors after kidney transplantation is associated with a higher relative risk of developing melanoma compared with other immunosuppressive therapies [2]. Understanding this heightened risk is critical for optimizing patient management and cancer prevention strategies in this vulnerable group.

Calcineurin inhibitors (CNIs) have been used for many years in patients undergoing solid organ transplantation and, according to current immunosuppressive guidelines for vascularized organ recipients, constitute the cornerstone of the most commonly applied lifelong immunosuppressive regimen after kidney transplantation. Kidney transplants account for nearly 60% of all transplant procedures in Poland [3]. The standard immunosuppressive regimen combines a calcineurin inhibitor—tacrolimus (TAC) or cyclosporine A (CsA)—with mycophenolic acid (MPA; administered as mycophenolate mofetil [MMF] or mycophenolate sodium [MPS]) and glucocorticosteroids (GS) [4,5,6].

The mechanism of action of CNIs and GS involves inhibition of T-cell activation through reduced cytokine production and suppression of clonal expansion, while MMF and MPS inhibit cellular proliferation. Despite differences in chemical structure, cyclosporine and tacrolimus share a similar mode of action, blocking the serine–threonine phosphatase calcineurin. Calcineurin is responsible for the dephosphorylation of nuclear factor of activated T cells (NF-AT), which in turn activates promoters of genes encoding key immunoregulatory cytokines, including interleukin 2 (IL-2), interleukin 4 (IL-4), interferon-γ (IFN-γ) and tumor necrosis factor-α (TNF-α) [7,8].

The aim of this triple-therapy regimen is to attenuate the recipient’s immune response to donor kidney antigens, thereby preventing graft rejection, maintaining stable graft function, and improving graft survival and patient quality of life [4,9,10]. This regimen provides one-year graft survival rates of 90–95% and acute rejection rates below 20% [4]. Nevertheless, long-term immunosuppressive therapy is associated with an increased risk of multiple complications, including infections, nephrotoxicity, metabolic disorders and therapy-related secondary malignancies [2,11].

In addition to its well-recognized clinical benefits, immunosuppression also increases the risk of virus-related malignancies such as lymphomas, cervical cancer, Kaposi sarcoma and hepatocellular carcinoma [12,13,14], as well as cancers unrelated to infections, including cutaneous melanoma (CM) and non-melanoma skin cancer (NMSC), the most common de novo malignancy after liver and kidney transplantation [15,16]. The mechanisms underlying increased carcinogenesis associated with CNI therapy have not yet been fully elucidated.

Advances in transplantation medicine and the expanding use of CNIs in non-transplant dermatologic conditions—such as atopic dermatitis, psoriasis, vitiligo or seborrheic dermatitis—highlight the need for a more thorough evaluation of cancer risk associated with this drug class. Existing studies exploring melanoma risk in relation to CNI therapy are scarce, and none have compared the oncologic safety profiles of individual CNIs in detail [17,18,19].

Despite an increasing number of international reports, large-scale epidemiological studies assessing this issue in Polish kidney transplant recipients are still lacking. Moreover, the relationship between the most frequently used immunosuppressive regimen (TAC or CsA + MPA + GS) and the risk of NMSC has not yet been comprehensively described in Poland. Understanding this relationship is essential for developing effective cancer prevention strategies, optimizing immunosuppressive therapy and improving patient education.

The aim of this study was to assess the overall incidence rate and cumulative incidence of CM in kidney transplant recipients treated with the standard triple immunosuppressive regimen consisting of a calcineurin inhibitor, MPA and GS. Additionally, we compared the risk of skin melanoma in patients treated with tacrolimus versus cyclosporine A in order to determine which drug may offer a more favorable oncologic safety profile.

To the best of our knowledge, this is the first study in Poland—and one of the largest so far published in Europe—designed to evaluate the association between CNI therapy and cutaneous melanoma risk in a large population of kidney transplant recipients, while simultaneously comparing the oncologic effects of TAC and CsA in this context.

## 2. Materials and Methods

### 2.1. Study Objectives

The primary objective of this study was to investigate whether the type of calcineurin inhibitor—specifically tacrolimus (TAC) or cyclosporine A (CsA)—administered to kidney transplant recipients receiving a CNI-based immunosuppressive regimen that also included mycophenolic acid (MPA, in the form of mycophenolate mofetil [MMF] or mycophenolate sodium [MPS]) and glucocorticosteroids (GS) influences the incidence of CM following kidney transplantation. Secondary objectives included comparing the cumulative incidence of CM between the TAC and CsA subgroups at follow-up intervals of 1–10 years post-transplantation, as well as constructing a comparative table to summarize incidence rates, cumulative events, and associated confidence intervals at these specified time points. Due to the small number of events, comparisons between TAC and CsA are exploratory and should be interpreted cautiously.

### 2.2. Statistical Analysis

The statistical analysis focused on estimating the incidence rate and cumulative incidence of CM following kidney transplantation, based on longitudinal data comprising the number of patients at risk and new events observed across annual periods post-transplantation. The analysis adhered to principles of survival analysis, specifically adapting the life table (actuarial) method to account for decreasing cohort size due to events, censoring, or loss to follow-up. No advanced modeling (e.g., Kaplan–Meier estimation or Cox proportional hazards) was applied, with emphasis on basic incidence metrics derived directly from the provided counts.

CM cases were identified using ICD-10 diagnostic codes recorded in administrative healthcare databases. As these databases do not contain detailed histopathological information, analyses were restricted to hospital-based diagnoses in order to minimize potential overreporting. In routine clinical practice, hospital discharge diagnoses of cutaneous melanoma typically correspond to cases confirmed by histopathological examination. Tumor-level pathological characteristics, including histological subtype, Breslow thickness, ulceration status, or disease stage, were not available. Diagnoses were not cross-validated with histopathological records from the National Cancer Registry.

Descriptive statistics were employed to characterize the study population and key variables. Continuous variables, such as age at diagnosis and time from transplantation to event, were summarized using the mean and standard deviation (SD). Count data, including the number of events and patients at risk, were reported as frequencies (n).

All hypothesis tests and confidence intervals were conducted at a significance level (alpha) of 0.05, corresponding to 95% confidence intervals and a threshold for statistical significance of *p* < 0.05.

### 2.3. Incidence Rate Estimation

Incidence rate (IR) for each interval was calculated as the ratio of new events to the number of patients at risk, scaled to 1000 person-years. This metric estimates the instantaneous risk of CM occurrence within each interval. Detailed formulas and computations for IR are provided in the Supplementary Materials.

### 2.4. Confidence Intervals for Incidence Rate

To quantify uncertainty in the incidence rate estimates, 95% confidence intervals (CIs) were calculated using the exact Poisson method, which is suitable for count data involving rare events. Incidence rates were expressed per 1000 person-years. For intervals with zero observed events, confidence intervals were reported as 0.00 for clarity of presentation. Full methodological details and formulae are provided in the Supplementary Materials.

### 2.5. Cumulative Incidence Estimation

Cumulative incidence was calculated as the proportion of the initial cohort that developed CM by the end of each follow-up, reflecting the overall disease burden over time. Estimates were based on cumulative event counts normalized to the initial cohort size.

### 2.6. Confidence Intervals for Cumulative Incidence Estimation

The 95% CIs for cumulative incidence were computed using the exact binomial (Clopper–Pearson) method for descriptive purposes. This approach treats cumulative events as binomial outcomes and does not explicitly account for time-dependent censoring; however, it is suitable for the descriptive aims of the analysis. Detailed computational procedures are described in the Supplementary Materials.

### 2.7. Risk Difference

The risk difference (RD) between the TAC and CsA subgroups was calculated as the absolute difference in cumulative incidence at each follow-up year. Relative risk estimation was not performed due to the absence of events in the CsA group. The 95% CI for RD was estimated using the Newcombe–Wilson hybrid method, which provides robust coverage in the presence of sparse data and zero events. Statistical significance was assessed using Fisher’s exact test with a one-sided alternative hypothesis assuming higher risk in the TAC group. Additional statistical details are presented in the Supplementary Materials.

### 2.8. Characteristics of the Statistical Tool

Analyses were conducted using the R Statistical language (version 4.5.2; [20]) on Windows 11 Pro 64-bit (build 26100), using the packages epitools (version 0.5.10.1; [21]), report (version 0.6.2; [22]), *dplyr* (version 1.1.4; [23]) and *knitr* (version 1.50; [24,25,26]).

## 3. Results

### 3.1. Demographics of the Studied Population

The study population consisted of 12,205 recipients of kidney transplants performed between 2010 and 2022. All patients received immunosuppressive therapy according to standard post-transplant protocols. Among them, 7107 patients were treated with a calcineurin inhibitor (CNI)-based regimen, including tacrolimus (TAC) or cyclosporine A (CsA), combined with mycophenolic acid (MMF/MPS) and glucocorticosteroids. Thus, the CNI-treated patients are a subset of the total study population.

Demographic analysis revealed a predominantly middle-aged population, with a mean age during follow-up of 47.97 years (standard deviation [SD] 1.15 years). In patients who developed cutaneous melanoma, the mean age at diagnosis was 61.21 years (SD 9.00 years). The mean interval from transplantation to CM diagnosis was 4.74 years (SD 2.18 years), emphasizing a delayed onset in this immunosuppressed group.

### 3.2. The Overall Incidence Rate and Cumulative Incidence of CM Following Kidney Transplantation

The data in Table 1 reveal a low overall incidence of CM among kidney transplant recipients, with a total of 27 cases observed in a population of 12,205 patients over 13 years of follow-up, corresponding to a final cumulative incidence of 0.23% (95% CI: 0.15–0.34). Annual incidence rates remained modest, ranging from 0.00 to 0.68 per 1000 person-years, with the highest rate noted in the seventh year post-transplantation. No events were recorded after the tenth year of follow-up. Comparisons with the general population are approximate and descriptive, as no age or sex standardization was performed, and should not be interpreted as formal relative risk estimates.

### 3.3. The Overall Incidence Rate and Cumulative Incidence of CM Following Kidney Transplantation Under CNI-Based Immunosuppressive Regimens

The analysis of CM incidence under CNI-based immunosuppressive regimens in kidney transplant recipients demonstrates an overall low risk profile over 10 years, with only nine cases observed in a cohort of 7107 patients, culminating in a cumulative incidence of 0.13% (95% CI: 0.06–0.24) (refer to Table 2). Incidence rates remained modest throughout, generally below 1 per 1000 person-years, though occasional fluctuations were noted, such as in year 4 (0.83; 95% CI: 0.23–2.13) and year 9 (1.60; 95% CI: 0.19–5.79). The absence of events in several years, particularly later in follow-up, reveals a possible plateau in risk amid declining cohort size due to attrition.

### 3.4. The Overall Incidence Rate and Cumulative Incidence of CM Following Kidney Transplantation Under CNI-Based Immunosuppressive Regimens Stratified by the Type of Calcineurin Inhibitor

The stratified analysis of CM incidence under CNI-based immunosuppressive regimens by calcineurin inhibitor type revealed a cumulative risk in the TAC subgroup, reaching 0.14% (95% CI: 0.06–0.26) by year 10, with all nine observed cases occurring exclusively in this group, while the CsA subgroup reported zero events throughout the follow-up period (Table 3). Among the nine incident CM cases, all occurred under CNI-based immunosuppressive regimen with TAC as the calcineurin inhibitor, with no cases in the CsA subgroup. The cases presented 2 to 9 years post-transplantation (mean latency approximately 5.6 years), predominantly in males (n = 8; 89%), and across age groups including two under 40 years, one in the 40–50 range, four in the 60–70 range, and two in the 70–80 range. The single female case was in the 70–80 age group at 6 years post-transplantation.

Annual incidence rates in TAC fluctuated between 0.00 and 1.72 per 1000 person-years, with notable peaks in years 4 and 9, whereas CsA rates remained at 0.00 across all intervals.

Risk differences progressively increased to 0.14% (95% CI: −0.20 to 0.26) by year 10 but were not statistically significant in any year (*p* ≥ 0.230).

To account for variability in the number of patients at risk across follow-up years, we additionally identified the years with the highest crude annual incidence in each cohort. Detailed comparisons are presented in Supplementary Table S1.

### 3.5. Melanoma-Free Survival in Kidney Transplant Recipients Under CNI-Based Immunosuppressive Regimen Stratified by the Type of Calcineurin Inhibitor

**Figure 1:**
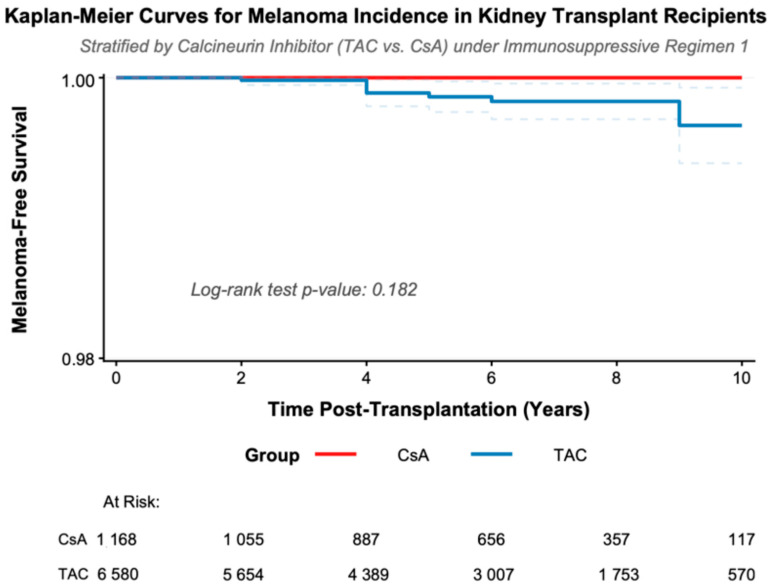
Kaplan–Meier estimates of melanoma-free survival in kidney transplant recipients stratified by calcineurin inhibitor therapy (tacrolimus vs. cyclosporine A).

The Kaplan–Meier curves in Figure 1 present melanoma-free survival over a 10-year follow-up in kidney transplant recipients treated with a CNI-based immunosuppressive regimen, stratified by calcineurin inhibitor type (TAC versus CsA). In the CsA group, melanoma-free survival remained constant at 1.0 throughout the observation period. In the TAC group, slight decreases in melanoma-free survival were observed, corresponding to a low absolute cumulative incidence of melanoma (<0.14%) over 10 years. The comparison between groups using the log-rank test yielded a *p*-value of 0.182.

## 4. Discussion

The standard immunosuppressive regimen after kidney transplantation consists of a calcineurin inhibitor (TAC/TAC MR/LCPT/CsA), an antiproliferative agent (MMF/MPS), and glucocorticosteroids. Tacrolimus is currently the most widely used CNI. This regimen ensures a 1-year graft survival rate of 90–95% and an acute rejection rate below 20%. The goals of immunosuppressive therapy are to maximize graft function, minimize rejection risk, and reduce treatment-related adverse effects [4,27,28].

Immunosuppressive management can be divided into three phases: induction, early (<3–6 months post-transplant), and late (>3–6 months). Long-term exposure to CNIs may increase the risk of skin cancer by promoting uncontrolled cell proliferation [29,30,31]. However, data on the relationship between CNI-based therapy and melanoma incidence in kidney transplant recipients have been lacking.

In the present study, we analyzed three parameters: the overall incidence rate and cumulative incidence of CM following kidney transplantation, the incidence of CM among recipients treated with a CNI-based regimen, and CM incidence stratified by the type of calcineurin inhibitor (tacrolimus vs. cyclosporine A).

Our analysis demonstrates a low overall incidence of malignant melanoma in a cohort of 12,205 kidney transplant recipients who underwent transplantation between 2010 and 2022. A total of 27 cases were identified over 13 years of follow-up, corresponding to a final cumulative incidence of 0.23% (95% CI: 0.15–0.34). The absence of new cases beyond year 10, with a cumulative incidence of 0.22%, may suggest a plateau in risk, although this trend may be influenced by attrition or competing events. When compared with the 10-year cumulative melanoma risk in the general Polish population (0.09%) [17], the difference appears notable but warrants further surveillance and research. This comparison is approximate and descriptive only, since no age or sex standardization was performed. Therefore, the observed difference should be interpreted with caution.

Among recipients treated with a CNI-based regimen, nine cases of melanoma were identified in a cohort of 7107 patients, yielding a cumulative incidence of 0.13% (95% CI: 0.06–0.24). Incidence rates remained low, with occasional fluctuations in years 4 and 9. These results support the favorable oncologic safety profile of CNI-based therapy; however, long-term dermatologic surveillance remains essential due to the potential for rare yet clinically significant complications.

Stratified analysis revealed that all melanoma cases occurred exclusively in the tacrolimus subgroup, resulting in a cumulative incidence of 0.14% (95% CI: 0.06–0.26) at year 10, whereas no cases were observed in the cyclosporine A subgroup. Approximately 90% of cases occurred in male recipients, predominantly in middle-aged and older adults, consistent with previously recognized demographic risk patterns [32,33,34]. Despite this marked distribution, risk differences between TAC and CsA were not statistically significant (*p* ≥ 0.230), reflecting the limited statistical power due to the rarity of events. The exclusive occurrence of cases in the TAC group may indicate a potential trend; however, confirmation requires larger, multicenter studies. The results suggest that the risk of developing melanoma in kidney transplant recipients treated with calcineurin inhibitors is very low, regardless of the specific drug used. The minimal differences observed between the TAC and CsA groups did not reach statistical significance, indicating that the choice between these two calcineurin inhibitors does not appear to have a substantial impact on melanoma incidence over the analyzed follow-up period.

This study has several important limitations. Detailed National Health Fund (NHF) registry data are available only from 2010 onward, limiting the duration of retrospective analysis. Potential diagnostic ICD misclassification or overreporting—particularly in primary care settings—cannot be excluded; hence, to minimize that, only hospital and specialist clinic data were included in this analysis. Registry-based datasets may differ in reporting methodology, which may contribute to discrepancies between NHF, Poltransplant, and the National Cancer Registry. Furthermore, the dataset lacks information on key individual risk factors such as UV exposure, skin phototype, occupational sun exposure, prior dermatologic conditions, family history, lifestyle factors, and the intensity or variability of immunosuppressive therapy. The low number of melanoma cases results in broad confidence intervals and limited statistical power.

Despite these limitations, the strengths of the study include its nationwide large sample size, rigorous inclusion criteria, and the use of high-quality national registry data. To our knowledge, this is the first study to examine melanoma incidence in such a large population of kidney transplant recipients in Poland and one of the largest in Europe. The results suggest that long-term CNI-based immunosuppression does not significantly increase melanoma risk, and the type of CNI does not exert a statistically significant effect. Nevertheless, continued dermatologic surveillance remains essential, particularly in the early post-transplant period, when most cases were identified.

## 5. Conclusions

In this large, nationwide analysis of kidney transplant recipients, we demonstrated that the overall incidence of cutaneous melanoma following kidney transplantation remains low, even in patients receiving long-term CNI-based immunosuppressive therapy. Although the cumulative 10-year risk of melanoma in this population exceeded that of the general Polish population, the absolute risk remained small and appears to have limited clinical impact. Similarly, the use of CNI-based regimens was not associated with a statistically significant increase in melanoma incidence compared with the overall transplant cohort.

Stratified analysis revealed that all melanoma cases occurred in the tacrolimus (TAC) subgroup, whereas no cases were reported among recipients treated with cyclosporine A (CsA). However, due to the rarity of events and limited statistical power of the analysis, these findings should be interpreted with caution and do not allow firm conclusions regarding differential melanoma risk between individual CNIs. This observation should therefore be regarded as hypothesis-generating and warrants further investigation in larger, prospectively designed studies.

During the 10-year follow-up, no significant differences in melanoma incidence were observed between kidney transplant recipients treated with tacrolimus and cyclosporine A. These findings suggest comparable safety of both treatment regimens with respect to melanoma risk.

Despite the low incidence observed, early recognition of melanoma remains clinically important. Regular dermatological surveillance—particularly in the early post-transplant period, when most cases were identified—should remain an integral component of post-transplant care. Patient education regarding self-examination and sun protection should also be emphasized, given the increased vulnerability of immunosuppressed individuals, especially older male recipients.

Several limitations of this study should be acknowledged. In addition to the inherent constraints of registry-based analyses, the potential contribution of CNI-related nephrotoxicity and kidney dysfunction to melanoma risk could not be assessed and should be considered in future research. It is possible that melanoma development in transplant recipients may be influenced not only by immunosuppressive therapy but also by chronic kidney disease-related factors.

Future studies incorporating detailed clinical data—such as renal function, immunosuppressive drug exposure, UV radiation, skin phototype, and lifestyle factors—will be necessary to further clarify melanoma risk and to refine individualized risk stratification strategies in kidney transplant recipients.

## Figures and Tables

**Table 1:** Incidence rate and cumulative incidence of CM following kidney transplantation over 13 years.

Follow-Up (Years)	Patients at Risk	New Events CM	Incidence Rate (per 1000 Person-Years) (95% CI)	Cumulative Events CM	Cumulative Incidence (%) (95% CI)
Kidney Tx	12,205	-	-	-	-
1	11,672	2	0.17 (0.02–0.62)	2	0.02 (0.00–0.06)
2	10,676	6	0.56 (0.21–1.22)	8	0.07 (0.03–0.13)
3	9787	1	0.10 (0.00–0.57)	9	0.07 (0.03–0.14)
4	8895	4	0.45 (0.12–1.15)	13	0.11 (0.06–0.18)
5	7869	3	0.38 (0.08–1.11)	16	0.13 (0.07–0.21)
6	6893	3	0.44 (0.09–1.27)	19	0.16 (0.09–0.24)
7	5867	4	0.68 (0.19–1.75)	23	0.19 (0.12–0.28)
8	4858	1	0.21 (0.01–1.15)	24	0.20 (0.13–0.29)
9	3919	2	0.51 (0.06–1.84)	26	0.21 (0.14–0.31)
10	2998	1	0.33 (0.01–1.86)	27	0.22 (0.15–0.32)
11	2082	0	0.00 (0.00–0.00)	27	0.22 (0.15–0.32)
12	1268	0	0.00 (0.00–0.00)	27	0.22 (0.15–0.32)
13	603	0	0.00 (0.00–0.00)	27	0.22 (0.15–0.32)

Note: Incidence rates are calculated assuming one-year intervals. Cumulative incidence is based on the initial population at risk.

**Table 2:** Overall incidence rate and cumulative incidence of CM in kidney transplant recipients receiving CNI-based immunosuppressive regimen.

Follow-Up (Years)	Patients at Risk	New Events	Incidence Rate (per 1000 Person-Years) (95% CI)	Cumulative Events	Cumulative Incidence (%) (95% CI)
Kidney Tx	7107	-	-	-	-
1	6896	0	0.00 (0.00–0.00)	0	0.00 (0.00–0.04)
2	6125	1	0.16 (0.00–0.91)	1	0.01 (0.00–0.08)
3	5465	0	0.00 (0.00–0.00)	1	0.01 (0.00–0.08)
4	4800	4	0.83 (0.23–2.13)	5	0.07 (0.02–0.16)
5	4044	1	0.25 (0.01–1.38)	6	0.08 (0.03–0.18)
6	3334	1	0.30 (0.01–1.67)	7	0.10 (0.04–0.20)
7	2614	0	0.00 (0.00–0.00)	7	0.10 (0.04–0.20)
8	1907	0	0.00 (0.00–0.00)	7	0.10 (0.04–0.20)
9	1247	2	1.60 (0.19–5.79)	9	0.13 (0.06–0.24)
10	627	0	0.00 (0.00–0.00)	9	0.13 (0.06–0.24)

*Note:* Incidence rates are calculated assuming one-year intervals. Cumulative incidence is based on the initial population at risk.

**Table 3:** Comparative incidence rate and cumulative incidence of CM in kidney transplant recipients under CNI-based immunosuppressive regimen by calcineurin inhibitor (TAC vs. CsA).

Follow-Up (Years)	TAC					CsA					Risk Difference (TAC vs. CsA) (%)	95% CI for RD (%)	*p*-Value
	Patients at Risk	New Events	Incidence Rate (per 1000 Person-Years) (95% CI)	Cumulative Events	Cumulative Incidence (%) (95% CI)	Patients at Risk	New Events	Incidence Rate (per 1000 Person-Years) (95% CI)	Cumulative Events	Cumulative Incidence (%) (95% CI)			
Kidney Tx	6580	-	-	-	-	1168	-	-	-	-	-	-	-
1	6401	0	0.00 (0.00–0.47)	0	0.00 (0.00–0.06)	1129	0	0.00 (0.00–2.66)	0	0.00 (0.00–0.26)	0.00	(−0.33 to 0.06)	1.000
2	5654	1	0.18 (0.00–0.99)	1	0.02 (0.00–0.08)	1055	0	0.00 (0.00–2.84)	0	0.00 (0.00–0.26)	0.02	(−0.31 to 0.09)	0.849
3	5032	0	0.00 (0.00–0.60)	1	0.02 (0.00–0.08)	966	0	0.00 (0.00–3.10)	0	0.00 (0.00–0.26)	0.02	(−0.31 to 0.09)	0.849
4	4389	4	0.91 (0.25–2.33)	5	0.08 (0.02–0.18)	887	0	0.00 (0.00–3.38)	0	0.00 (0.00–0.26)	0.08	(−0.25 to 0.18)	0.442
5	3668	1	0.27 (0.01–1.52)	6	0.09 (0.03–0.20)	782	0	0.00 (0.00–3.83)	0	0.00 (0.00–0.26)	0.09	(−0.24 to 0.20)	0.375
6	3007	1	0.33 (0.01–1.85)	7	0.11 (0.04–0.22)	656	0	0.00 (0.00–4.57)	0	0.00 (0.00–0.26)	0.11	(−0.23 to 0.22)	0.318
7	2359	0	0.00 (0.00–1.27)	7	0.11 (0.04–0.22)	514	0	0.00 (0.00–5.83)	0	0.00 (0.00–0.26)	0.11	(−0.23 to 0.22)	0.318
8	1753	0	0.00 (0.00–1.71)	7	0.11 (0.04–0.22)	357	0	0.00 (0.00–8.39)	0	0.00 (0.00–0.26)	0.11	(−0.23 to 0.22)	0.318
9	1163	2	1.72 (0.21–6.21)	9	0.14 (0.06–0.26)	208	0	0.00 (0.00–14.40)	0	0.00 (0.00–0.26)	0.14	(−0.20 to 0.26)	0.230
10	570	0	0.00 (0.00–5.25)	9	0.14 (0.06–0.26)	117	0	0.00 (0.00–25.60)	0	0.00 (0.00–0.26)	0.14	(−0.20 to 0.26)	0.230

In year 1 (RD = 0.00%), the CI (−0.33% to 0.06%) is appropriately shifted, centering slightly off zero because of the smaller CsA sample size inflating variance on one side.

## Data Availability

The data analyzed in this study were obtained from the National Health Fund administrative databases and are not publicly available due to legal and privacy restrictions. Access to the data is subject to approval by the National Health Fund and applicable national data protection regulations.

## Supplementary Materials

The following supporting information can be downloaded at https://www.mdpi.com/article/10.3390/cancers18040642/s1: detailed description of mathematical calculation of the statistical section; Table S1: Top Three Years of Highest Incidence Rates of Cutaneous Melanoma by Population/Cohort.

## List of Abbreviations

CI	Confidence interval
CIt	Cumulative incidence for interval t
CM	Cutaneous melanoma
CsA	Cyclosporine A
CNI	Calcineurin inhibitor
GS	Glucocorticosteroids
IFN-γ	Interferon gamma
IL-2	Interleukin 2
IL-4	Interleukin 4
IRt	Incidence rate for interval t
MPA	Mycophenolic acid (administered as mycophenolate mofetil [MMF] or mycophenolate sodium [MPS])
NF-AT	Nuclear factor of activated T cells
NMSC	Non-melanoma skin cancer
PNCR	Polish National Cancer Registry
PNHF	Polish National Health Fund
RD	Risk difference
RR	Relative risk
SIR	Standardized incidence ratio
TAC	Tacrolimus
TNF-α	Tumor necrosis factor alpha
