# Supplementary material for: Melanoma Risk in 12,205 Kidney Transplant Recipients Receiving Calcineurin Inhibitor-Based Immunosuppression: A Nationwide Analysis of Polish National Health Fund Data (2010–2022)
Source: Cancers (Basel). 2026 Feb 16;18(4):642. doi: 10.3390/cancers18040642 (PMC12939293; doi:10.3390/cancers18040642)
Supplement: Supplementary file 1 [file cancers-18-00642-s001.zip › cancers-4107875-supplementary.pdf]

Supplementary

Incidence rate estimation

The incidence rate (IR_t) for each interval t was calculated as:

$$IR_t = \left(\frac{e_t}{n_t}\right) \times 1000 \ (S1),$$

where: e_t – number of new events in interval t, n_t – number of patients at risk at the start of interval t. This equation (S1) assumes that events and censoring occur uniformly across the interval, providing a conservative estimate for rare outcomes like CM. Rates were computed iteratively for each t.

Confidence intervals for incidence rate

To quantify uncertainty in the incidence rate estimates, 95% confidence intervals (CIs) were calculated using the exact Poisson method, which is suitable for count data involving rare events. This approach treats the number of new events e_t as following a Poisson distribution with mean $\lambda_t = IR_t \times n_t/1000$, where the exposure is approximated by n_t (person-years).

The exact 95% CI for the Poisson parameter λ_t is derived by solving for the bounds where the cumulative Poisson probabilities equal 0.025 and 0.975, respectively, based on the observed et. Specifically, the lower bound λ_L satisfies:

$$\sum_{k=0}^{e_t} \frac{e^{-\lambda_L} \lambda_L^k}{k!} = 0.975 \ (S2.1),$$

and the upper bound λ_U satisfies:

$$\sum_{k=0}^{e_t} \frac{e^{-\lambda_U} \lambda_U^k}{k!} = 0.025 \ (S2.2),$$

These bounds are then scaled to the incidence rate per 1000 person-years:

$$CI_{IR_t} = \left(\frac{\lambda_L}{n_t/_{1000}}, \ \frac{\lambda_U}{n_t/_{1000}}\right) \ (S2.3)$$

For intervals with zero events (e_t=0), the CI was simplified to (0.00, 0.00) for presentation, although the exact upper bound is non-zero in precise computations.

Cumulative incidence estimation

Cumulative incidence (CI_t) represents the proportion of the initial cohort that developed CM by the end of interval *t*, serving as a measure of overall disease burden over time. It was derived by first computing cumulative events ($E_t = \sum_{i=1}^{t} e_i$) and then normalizing by the initial cohort size.

$$E_t = \sum_{i=1}^{t} e_i \text{ (S3.1)},$$

$$CI_t = \left(\frac{E_t}{N}\right) \times 100 \text{ (S3.2)},$$

where: E_t – Cumulative number of events up to interval *t*, N – initial number of patients at risk.

Confidence intervals for cumulative incidence estimation

The 95% CIs for cumulative incidence were computed using the exact binomial method (Clopper-Pearson interval), approximating the cumulative proportion as a binomial outcome for descriptive purposes. This treats the cumulative events E_t as successes in *N* independent trials, with proportion $p_t = E_t/N$

The exact 95% CI for p_t is obtained by inverting the binomial cumulative distribution function, solving for the bounds where: lower bound p_L satisfies

$$\sum_{k=E_t}^{N} \binom{N}{k} p_L^k (1 - p_L)^{N-k} = 0.025 \text{ (S4.1)},$$

upper bound p_U satisfies

$$\sum_{k=0}^{E_t} \binom{N}{k} p_U^k (1 - p_U)^{N-k} = 0.025, \text{ (S4.2)},$$

These bounds are then scaled to percentages:

$$CI_{CI_t} = (p_L \times 100, p_U \times 100) \text{(S4.3)},$$

This method does not incorporate time-dependent censoring, potentially introducing minor bias in long-term estimates, but is appropriate for the descriptive nature of the analysis.

Risk difference

Mathematically, RD was defined as:

$$RD = P_{TAC} - P_{CsA} = \frac{e_{TAC}}{N_{TAC}} - \frac{e_{CsA}}{N_{CsA}} \text{ (S5)}$$

where e_{TAC} and e_{CsA} represent the cumulative number of CM events in the TAC and CsA groups, respectively, and N_{TAC} and N_{CsA} denote the initial cohort sizes for each subgroup.

The Wilson score interval for a proportion $p=e/n$ is given by:

$$p \pm \frac{z\sqrt{\frac{p\,(1-p)}{n} + \frac{z^2}{4n^2}}}{(1+\frac{z^2}{n})} \text{ (S6),}$$

where z=1.96 for a 95% CI.

The RD 95% CI is then derived as:

$$95\%CI = RD \pm \sqrt{Var(P_{TAC}) + Var(P_{CsA})} \text{ (S7),}$$

with variances approximated from the respective Wilson intervals, ensuring asymmetry and boundary respect without requiring continuity corrections.

The test statistic is based on the hypergeometric distribution for the 2×2 table, computed as:

$$p = \sum_{k=e_{TAC}}^{\min(e_{TAC}+N_{TAC}, e_{TAC}+e_{CsA})} \frac{\binom{N_{TAC}}{k}\binom{N_{CsA}}{e_{TAC}+e_{CsA}-k}}{\binom{N_{TAC}+N_{CsA}}{e_{TAC}+e_{CsA}}} \text{ (S8)}$$

Table S1. Top Three Years of Highest Incidence Rates of Cutaneous Melanoma by Population/Cohort.

Population/Cohort	Rank	Follow-up (Years)	Patients at Risk	New Events	Incidence Rate[1]	95% CI
Overall Kidney Tx	1	7	5,867	4	0.68	0.19–1.75
Overall Kidney Tx	2	2	10,676	6	0.56	0.21–1.22
Overall Kidney Tx	3	9	3,919	2	0.51	0.06–1.84
CNI-based	1	9	1,247	2	1.60	0.19–5.79
CNI-based	2	4	4,800	4	0.83	0.23–2.13
CNI-based	3	6	3,334	1	0.30	0.01–1.67
TAC	1	9	1,163	2	1.72	0.21–6.21
TAC	2	4	4,389	4	0.91	0.25–2.33
TAC	3	6	3,007	1	0.33	0.01–1.85
CsA	-	1	1,129	0	0.00	0.00–2.66
CsA	-	2	1,055	0	0.00	0.00–2.84
CsA	-	3	966	0	0.00	0.00–3.10

Note: [1] - per 1,000 Person-Years. Years are ranked by descending incidence rate within each cohort. For the CsA subgroup, where all rates are zero, the first three years are presented for completeness, without assigned ranks. Incidence rates assume one-year intervals and are derived from the original data using the exact Poisson method for confidence intervals.
